# SIRT3 promotion reduces resistance to cisplatin in lung cancer by modulating the FOXO3/CDT1 axis

**DOI:** 10.1002/cam4.3728

**Published:** 2021-03-02

**Authors:** Yang Cao, Ping Li, Haicun Wang, Lei Li, Quanwang Li

**Affiliations:** ^1^ The Third Department of Medical Oncology the Third People's Hospital of Zhengzhou Zhengzhou P. R. China; ^2^ Medical Department Women & Infants Hospital of Zhengzhou Zhengzhou P. R. China; ^3^ Department of Oncology Dongfang Hospital of Beijing University of Chinese Medicine Beijing P. R. China

**Keywords:** chromatin licensing and DNA replication factor 1, cisplatin, Forkhead box O3a, lung cancer, sirtuin 3

## Abstract

**Background:**

Cisplatin is an extensively used chemotherapy agent for lung cancer, but its drug resistance serves as a huge obstacle for chemotherapy failure of lung cancer patients. Hence, researchers aimed to determine role of sirtuin 3 (SIRT3) considering its action in cisplatin resistance of lung cancer.

**Methods:**

The expression patterns of SIRT3, FOXO3, and CDT1 were determined using RT‐qPCR and Immunoblotting in lung cancer. Immunofluorescence and Co‐IP were adopted to detect co‐localization and interaction of FOXO3 and CDT1. Loss‐ and gain‐function assays were conducted to determine roles of SIRT3, FOXO3, and CDT1 in resulting pathological changes, while biological behavior of cells was determined using a combination of CCK‐8, flow cytometry, colony formation, and Transwell assays. The effects of SIRT3 and CDT1 were determined in the nude mice xenografted with the tumor. The proliferation‐, angiogenesis‐, and apoptosis‐associated factors levels were determined using Immunoblotting.

**Results:**

SIRT3, FOXO3, and CDT1 expression was suppressed in the lung cancer tissues and cells. FOXO3 positively regulates the CDT1 expression pattern and SIRT3 elevation inhibits FOXO3 at the acetylated level, thus, elevating FOXO3 expression. The elevation of SIRT3, FOXO3, or CDT1 inhibited cell cisplatin resistance of lung cancer cells as well as inhibited viability, proliferation, and invasion *in vitro*. In vivo experiments, SIRT3 depletion elevated Ki‐67 and VEGFA levels, but downregulated cleaved caspase 3 level.

**Conclusion:**

Collectively, overexpressed SIRT3 elevates expression of FOXO3a/CDT1 axis, thus, contributing to enhanced sensitivity of lung cancer cells.

## Graphical abstract

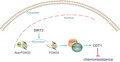

## 1 INTRODUCTION

Lung cancer serves as most frequently diagnosed cancer (11.6%) with the highest cancer‐associated death for both sexes worldwide.^1^ Chemotherapeutic agents or chemotherapy are gold standard treatment modality for non‐small cell lung cancer (NSCLC).^2^, ^3^ Cisplatin is the most extensively used chemotherapy agent for lung cancer.^4^ However, the cisplatin‐induced resistance has emerged as a massive obstacle for chemotherapy failure of lung cancer patients, and consequently provokes higher malignancy and metastasis.^5^ Thus, elucidating the underlying mechanism involved in cisplatin resistance of lung cancer cells holds key to attenuating and improving the efficacy for lung cancer.

Chromatin licensing and DNA replication factor 1 (CDT1) is a fundamental protein to replicate the corresponding origin licensing in the G1 phase, and would be degraded during S phase but re‐accumulate in G2 phase.^6^ The accumulation of CDT1 concentration regulates DNA replication phenotype attributed to the loss of function of the NEDD8‐activating enzyme (NAE), thus, affecting the re‐replication and apoptosis in human tumor cells.^7^ However, upstream targets of CDT1 in lung cancer remains unidentified. Forkhead box O3a (FOXO3a or FOXO3) has could serve as a regulator in the cell cycle progression by inhibiting CDT1 ubiquitination to further stabilize its expression.^8^ FOXO3a regulates various stress responses upon oxidative stress, hypoxia, and DNA damage, thus, serving as a crucial participant in cellular homeostasis, stress response, and longevity.^9^ In regard to the effect of FOXO3a on drug resistance, numerous reports have elucidated its vital function on EGFR mutation‐independent gefitinib resistance of lung cancer.^10^ Interestingly, an existing study documented the ability of sirtuin 3 (SIRT3) to regulate FOXO3a in prostate cancer.^11^ SIRT3, a class III lysine deacetylase, is principally localized in mitochondria and modulates mitochondrial respiration and oxidative stress resistance enzymes.^12^ The role of SIRT3 and FOXO3a has also been revealed in small‐cell lung cancer.^13^ On the basis of the aforementioned data, we hypothesized that SIRT3 might regulate cisplatin resistance of lung cancer cells *via* FOXO3a/CDT1 axis. In this study, we performed gain‐ and loss‐of‐function analysis to elucidate molecular mechanism regarding the involvement of cisplatin resistance of lung cancer cells.

## 2 MATERIALS AND METHODS

### 2.1 Ethics statement

Subjects were included using informed consent for a protocol granted by Ethics committee of our hospital. All clinic operations followed principles embodied in *Declaration of Helsinki*. Animal experiments were performed in accordance with ethical guidelines for animal experiment systems approved by Animal Committee of our hospital.

### 2.2 Samples

The lung cancer tissues and its matched adjacent normal tissues were obtained from 50 patients who underwent radical surgery of lung cancer in our hospital from June 2017 to June 2018 (28 males and 22 females). The adjacent normal tissues were non‐lesion tissues resected during operation and confirmed by pathological diagnosis.

### 2.3 Cell culture and infection

Lung cancer cells (H460, A549, and HCC1588) and normal lung cells (MRC5) were cultured in DMEM/F‐12 medium (11330057, Gibco), and added with 10% fetal bovine serum (FBS; 16000044, Gibco), 100 µg/ml streptomycin, and 100 U/ml penicillin. Next, cells were cultured in an incubator (37°C, 5% CO_2_), and 95% saturated humidity (90% medium + 10% FBS + 1% streptomycin, and penicillin), respectively. The cells were passaged at density of about 80%.

Cells were seeded in 6‐well plates at a density of 3 × 10^5^ cells/well, and infected with lentiviral vectors containing oe‐CDT1, oe‐NC, sh‐FOXO3‐1, sh‐FOXO3‐2, oe‐SIRT3, sh‐SIRT3 + oe‐NC, sh‐NC + oe‐NC, sh‐SIRT3 + oe‐CDT1, sh‐FOXO3 + DMSO, or sh‐FOXO3 + MG132 (Proteasome inhibitor), after cell growth density reached 50%. The before‐mentioned lentiviruses were synthesized by Genechem Co., LTD. The transfection efficiency was tested by RT‐qPCR, and 1 µg/ml puromycin was adopted to screen stably transduced cell lines.

### 2.4 RT‐qPCR

Total RNA was extracted using Trizol (15596026, Invitrogen) and RNA was reversely transcribed into cDNA using Reverse Transcription Kit (RR047A, Takara) adhering to manufacture protocol. The sample was loaded using a SYBR Premix EX Taq kit (RR420A, Takara), and subjected to real time PCR in a real‐time PCR instrument (ABI7500, ABI). The relative expression level of mRNA was normalized to glyceraldehyde‐3‐phosphate dehydrogenase (GAPDH) and was calculated using 2‐ΔΔCt method. Primers were synthesized in Sangon (Table 1).

**TABLE 1:** Primer sequences used for RT‐qPCR.

Target	Primer sequences (5′−3′)
FOXO3	F: 5′‐CAAAATAGCTACTTACCTTTGCAGAT‐3′
	R: 5′‐CAACAAACGCTAGAAAAGGAGA‐3′
GAPDH	F: 5′‐GAAGGTGAAGGTCGGAGTC‐3′
	R: 5′‐GAAGATGGTGATGGGATTTC‐3′
SIRT3	F: 5′‐ACCCAGTGGCATTCCAGAC‐3′
	R: 5′‐GGCTTGGGGTTGTGAAAGAAG‐3′

### 2.5 Immunoblotting

Total protein was collected and lysed using Radio‐Immunoprecipitation Assay lysate (Beyotime) supplemented with phenylmethylsulfonyl Fluoride. The protein concentration was tested using a bicinchoninic acid protein quantification kit. The proteins were incubated on ice for 30 min, centrifuged at 8000 *g* for 10 min at 4°C, and supernatant was collected. Next, 50 µg of protein was taken and dissolved in 2× SDS loading buffer. After boiling at 100°C for 5 min. Next, Protein were separated by SDS‐PAGE and transferred to PVDF membrane. The membrane was blocked with 5% skim milk powder at room temperature for 1 h. Then, membrane was probed overnight at 4°C with diluted primary antibodies CDT1 (1: 1000, ab83174, Abcam), Ki‐67 (1: 500, ab15580, Abcam), V‐EGFA (1: 1000, ab46154, Abcam), FOXO3 (1: 1000, ab70315, Abcam), acetylated (Ace)‐FOXO3 (1: 1000, ab70315, Abcam), flag (1: 2000, ab1170, Abcam), SIRT3 (1: 1000, ab86671, Abcam), cleared caspase3 (1: 1000, ab2302, Abcam), and β‐actin (internal reference; ab8227, 1: 2500, Abcam). The membrane was reprobed with horseradish peroxidase labeled goat anti rabbit secondary antibody Immunoglobulin G (IgG) H&L (ab205719, Abcam) for 1 h at room temperature. Then, membrane was incubated with chemiluminescence fluorescence detection kit (Cat. No. BB‐3501, Amersham). The membrane was photographed using Bio‐Rad image analysis system (Bio‐Rad Laboratories) and gray scale of image was analyzed using Quantity One v4.6.2 software with β‐actin as control.

### 2.6 Immunofluorescence

The specimen was fixed using 10% formaldehyde, paraffin‐embedded, and sectioned (4 µm thick). Sections were placed in a 60°C oven for 1 h, dewaxed with xylene, dehydrated with gradient alcohol, incubated in 3% H_2_O_2_ (Sigma) at 37°C for 30 min, and boiled at 0.01 M citrate buffer at 95°C for 20 min before sections were cooled. The sections were blocked by normal goat serum working solution at 37°C for 10 min, and added with primary antibody dropwise (mixture of FOXO3 and CDT1), followed by incubation at 4°C overnight. Next, sections were probed with secondary antibody for 1 h. The cell nucleus was counterstained with 1 µg/ml 4,6‐diamidino‐2‐phenylindole at room temperature for 4 min. The sections were observed under a microscope after 10% glycerol/PBS mounting, and then, independently scored by two people in double‐blinded fashion.

### 2.7 Flow cytometry

The next day after transfection, cells were digested with 0.25% trypsin, and digestion was terminated with DMEM medium containing 10% fetal bovine serum. The cells were centrifuged at 1000 r/min for 5 min, and supernatant was discarded. The cells were fixed with 70% ethanol precooled at 4°C, before cell concentration was adjusted to 1 × 10^6^ cells/ml. Next, cells were added with Annexin V‐fluorescein isothiocyanate (FITC)/propidium iodide (PI) (556547, Shuojia Biotechnology) to stain for 10 min, and placed in 4°C refrigerator for 15–30 min. The cells were put into a flow cytometer (XL type, Coulter, Inc.). To measure cell apoptosis, fluorescence of FITC and PI was activated at 488 nm wavelength and detected using 525 nm and 620 nm filter, respectively. The apoptosis rate was expressed as percentage of apoptotic cells in total number of cells.

### 2.8 Cell counting kit‐8 (CCK‐8)

After 48‐h transfection, cells were digested and seeded onto 96‐well plates (2500 cells/well). The cells were added with 10 µl of CCK‐8 solution (CK04‐1000, Dojindo) during detection. After incubating for 2 h in incubator, plate was placed in a microplate reader to assess optical density (OD) value at 450 nm wavelength. The statistical analysis was conducted on 8 wells in each group, and a growth curve was drawn accordingly.

### 2.9 Colony formation assay

The cells were seeded onto 6‐well plate (approximately 3000 cells/well) following cell digestion. After incubation (37°C; 14 d), cells were fixed with 20% methanol and stained with 0.1% crystal violet, and formed colonies were counted.

### 2.10 Transwell assay

The upper surface of membrane in Transwell chamber was covered with Matrigel (BD Biosciences) and incubated at 37°C for 30 min to facilitate the polymerization of Matrigel into gel following basal membrane hydration. The cells were cultured in serum‐free medium for 12 h, harvested, and resuspended with serum‐free medium (1 × 10^5^ cells/mL). Medium containing 10% fetal bovine serum was added to lower chamber, and 100 µl of cell suspension was added to Transwell chamber and incubated. The noninvaded cells were wiped off using a cotton swab, fixed with 100% methanol, and stained with 1% toluidine blue (Sigma). The number of invasive cells was counted manually in five randomly selected areas under an inverted light microscope (Carl Zeiss).

### 2.11 Co‐immunoprecipitation (Co‐IP)

A549 cells under specific treatment were cultured for 48 h, and whole cell lysate was prepared with Triton lysis buffer. Anti‐Flag M2 (Sigma) and anti‐c‐Myc mouse monoclonal antibody (9B11; Cell Signaling) were adopted for Co‐IP assay. The protein was eluted by boiling in sodium dodecyl sulfate (SDS) sample buffer and determined by Immunoblotting.

### 2.12 Tumor xenografts in nude mice

A total of 96 BALA/C nude mice (4–6 weeks old, weighed 18–25 g) were obtained from Hunan SJA Laboratory Animal Co., Ltd. and were housed in cages in Specific Pathogen Free (SPF) animal laboratory (humidity of 60%~65%; temperature of 22°C~25°C) with free access to food and water under 12 h light and dark cycles. After 1‐week adaptive feeding, nude mice were injected with A549 cells treated with lentiviral vectors containing oe‐NC, oe‐CDT1, control, sh‐NC, sh‐CDT1, SIRT3 + oe‐NC or sh‐SIRT3 + oe‐CDT1 (12 mice/group). Briefly, after washing twice with PBS, 1 × 10^6^ stably constructed cells were resuspended in 50 µl of physiological saline, added with 50 µl of Matrigel Matrix, mixed, and mixture was subcutaneously injected into nude mice. Subsequently, tumor volume at each time point was observed, recorded, and calculated using formula: V = (A × B2)/2 (A represents long diameter, B, short diameter, and unit is mm^3^). Twenty days later, nude mice were sacrificed by carbon dioxide asphyxiation, tumors were harvested, weighed, and photographed.

### 2.13 Statistical analysis

All data were processed and analyzed using SPSS 21.0 statistical software (IBM Corp.). Measurement data were summarized by mean ± standard deviation of at least three independent tests. The unpaired *t*‐test was applied for comparisons between two groups. One‐way analysis of variance (ANOVA) was used for comparisons among more than two groups, followed by Tukey's post hoc test. The significance level of difference between results was established as 0.05.

## 3 RESULTS

### 3.1 CDT1 expression was poorly expressed in lung cancer

**FIGURE 1:**
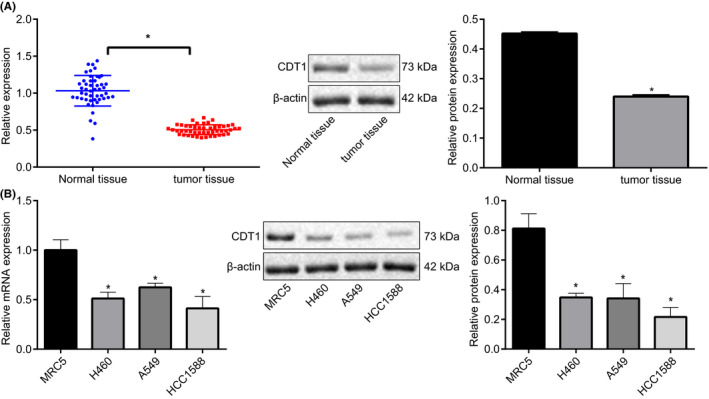
CDT1 expression was suppressed in lung cancer tissues and cells. (A) The mRNA and protein CDT1 expression in lung cancer tissues and adjacent normal tissues assessed by RT‐qPCR and Immunoblotting (n = 50). (B) The mRNA and protein CDT1 expression in lung cancer cells (H460, A549, and HCC1588) and normal lung cells (MRC5) assayed by RT‐qPCR and Immunoblotting. *p < 0.05 vs. adjacent normal tissues or MRC5 cells. The unpaired *t*‐test was applied for comparisons between two groups

We adopted RT‐qPCR and Immunoblotting in Figure 1A, CDT1 expression was markedly inhibited in lung cancer tissues relative to adjacent normal tissue. To further validate its expression, RT‐qPCR and Immunoblotting were conducted to assess CDT1 expression in lung cancer cells (H460, A549, and HCC1588) and normal lung cells (MRC5), revealing that compared with MRC5 cells, CDT1 levels were strikingly suppressed in H460, A549, and HCC1588 cells, and A549 cells with most downregulated CDT1 level were adopted for subsequent experiments (Figure 1B). The aforementioned data were indicative of a poor CDT1 expression pattern in both lung cancer tissues and cells.

### 3.2 CDT1 overexpression inhibits tumor progression and angiogenesis of A549 cells

**FIGURE 2:**
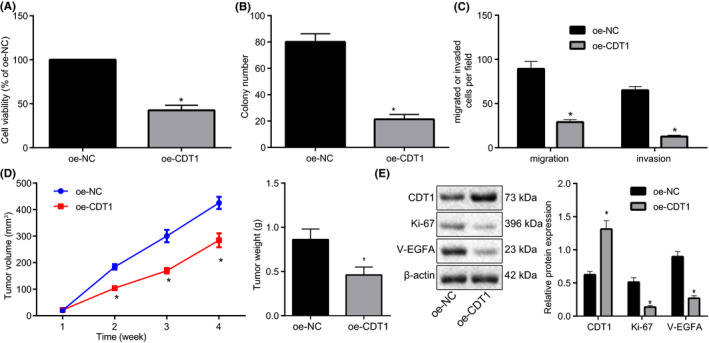
CDT1 elevation suppresses tumor progression and angiogenesis of A549 cells. (A) The viability of A549 cells following CDT1 overexpression determined using CCK‐8. (B) The proliferation of A549 cells following CDT1 overexpression assessed using colony formation assay. (C) The invasion of A549 cells following CDT1 overexpression determined using Transwell. (D) The tumor volume and weight extracted from nude mice xenografted with tumor following CDT1 overexpression. (E) The protein expression of CDT1, Ki‐67, and VEGFA determined using Immunoblotting. *p < 0.05 vs. A549 cells transduced with lentiviral vectors containing oe‐NC or mice injected with A549 cells treated with lentiviral vectors containing oe‐NC. The unpaired *t*‐test was applied for comparisons between two groups

We overexpressed CDT1 expression in A549 cells. The overexpression efficiency was validated using RT‐qPCR (Figure 2E). Next, 1 µg/ml puromycin was adopted to screen stably transduced cell lines. CCK‐8, colony formation assay and Transwell (Figure 2A–C) exhibited that CDT1 overexpression markedly reduced viability, proliferation, and invasion of A549 cells compared with oe‐NC treatment.

To better elucidate its role *in vivo*, we injected nude mice with stably constructed A549 cells treated with lentiviral vectors containing oe‐NC or oe‐CDT1. The tumor volume and weight were recorded. As expected, strikingly reduced tumor volume and weight were observed in response to oe‐CDT1 compared with oe‐NC (Figure 2D). Subsequently, levels of proliferation‐related antigen Ki‐67 and angiogenesis‐related factor VEGFA were determined using Immunoblotting, demonstrating markedly declined Ki‐67 and VEGFA levels following CDT1 overexpression (Figure 2E).

### 3.3 FOXO3 elevation promoted sensitivity to cisplatin of lung cancer cells by upregulating CDT1

**FIGURE 3:**
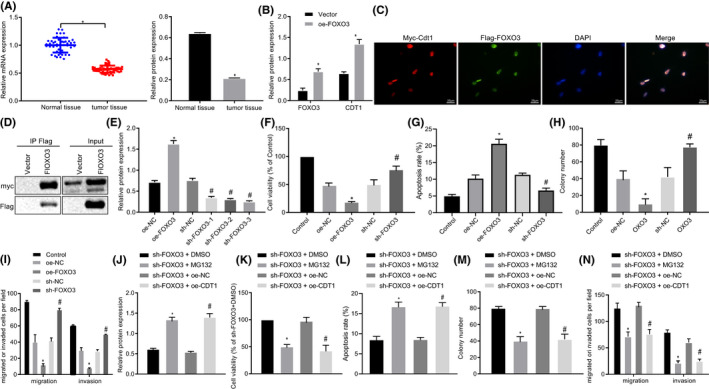
FOXO3 silencing promoted cisplatin resistance to lung cancer cells, which was reversed by CDT1 elevation. (FOXO3 was upregulated or downregulated in A549 cells in Panel F–I, while FOXO3‐knocked down cells were treated with overexpressed CDT1 or proteasome inhibitor MG132 in Panel K–M). (A) The mRNA and protein FOXO3 expression in lung cancer tissues and adjacent normal tissues assessed by RT‐qPCR and Immunoblotting (n = 50). (B) The protein expression of FOXO3 and CDT1 following FOXO3 overexpression determined by Immunoblotting. (C) The co‐localization of FOXO3 and CDT1 identified by Immunofluorescence. (D) The interaction between FOXO3 and CDT1 detected by Co‐IP (400×). (E) The transfection efficiency of FOXO3 validated using Immunoblotting. (F) The viability of A549 cells determined using CCK‐8. (G) The apoptosis of A549 cells determined using flow cytometry. (H) The proliferation of A549 cells assessed using colony formation assay. (I) The invasion of A549 cells following CDT1 overexpression determined using Transwell. (J) The protein level of CDT1 after MG132 treatment validated by Immunoblotting. (K) The viability of A549 cells determined using CCK‐8. (L) The apoptosis of A549 cells determined using flow cytometry. (M) The proliferation of A549 cells assessed using colony formation assay. (N) The invasion of A549 cells following CDT1 overexpression determined using Transwell. *p < 0.05 vs. adjacent normal tissues, oe‐NC or sh‐FOXO3 + DMSO; #*p* < 0.05 vs. sh‐NC or sh‐FOXO3 + oe‐NC. The unpaired *t*‐test was applied for comparisons between two groups. One‐way ANOVA was used for comparisons among more than two groups, followed by Tukey's post hoc test

RT‐qPCR and Immunoblotting displayed that FOXO3a expression was strikingly suppressed in lung cancer tissues compared with adjacent normal tissue (Figure 3A). Subsequently, we treated A549 cells with Flag‐FOXO3. Immunoblotting results identified expression of CDT1 potently rose after overexpressing FOXO3 (Figure 3B). To determine subcellular localization of FOXO3 and CDT1, we co‐transfected A549 cells with Myc‐CDT1 and Flag‐FOXO3 vectors. Immunofluorescence results displayed that FOXO3 and CDT1 were red and green in nucleus, respectively, and fluorescence was converted to yellow when fluorescence was overlapped, suggesting that they were co‐localized in nucleus (Figure 3C). Meanwhile, we conducted Co‐IP. As expected, Myc‐CDT1 were interacted with Flag‐FOXO3 in lung cancer cells (Figure 3D).

In subsequent analysis, to elucidate whether FOXO3/CDT1 axis was related to resistance of cisplatin to lung cancer, FOX3 was downregulated and upregulated in A549 cells, and then, treated with cisplatin. The transfection efficiency was validated using Immunoblotting and sh‐FOXO3‐1 with most significantly silenced FOXO3 expression was selected in subsequent experiments (Figure 3E). A series of procedures including CCK‐8, flow cytometry, colony formation assay, and Transwell were adopted to determine viability, apoptosis, proliferation, and invasion. Results demonstrated that overexpression of FOXO3 reduced viability, proliferation and invasion, but promoted apoptosis of cells after cisplatin treatment compared with sh‐NC treatment, but sh‐FOXO3 treatment promoted resistance to cisplatin‐induced decline in viability, proliferation, and invasion but increase in apoptosis of cells (Figure 3F–I).

Further, we treated FOXO3‐knocked down cells with overexpressed CDT1 or proteasome inhibitor MG132. Immunoblotting results verified that MG132 treatment elevated protein level of CDT1 inhibited by sh‐FOXO3 treatment (Figure 3J). After cisplatin treatment, cells treated with sh‐FOXO3 + MG132 or sh‐FOXO3 + oe‐CDT1 exhibited inhibited cell viability, proliferation, and invasion, but promoted apoptosis and sensitivity to cisplatin, while an opposite trend was observed in cells upon sh‐FOXO3 + DMSO or sh‐FOXO3 + oe‐NC treatment (Figure 3K–N). Coherently, FOXO3 promoted cisplatin resistance to lung cancer cells, which was reversed by CDT1 elevation.

### 3.4 SIRT3 elevation inhibits FOXO3 at acetylated level, thus, elevating FOXO3 expression

**FIGURE 4:**
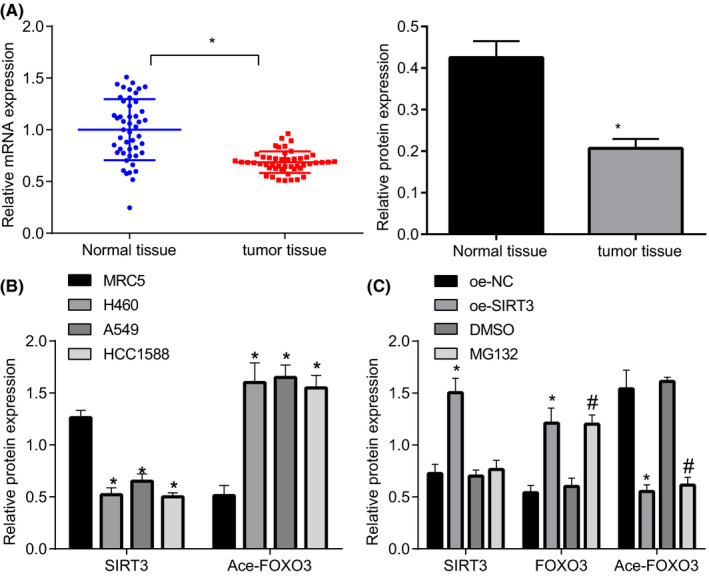
SIRT3 inhibited FOXO3 at acetylated level, thus, suppressing FOXO3 expression. (A) The mRNA and protein SIRT3 expression in lung cancer tissues and adjacent normal tissues assessed by RT‐qPCR and Immunoblotting (n = 50). (B) The SIRT3 protein expression and ace‐FOXO3 level in lung cancer cells (H460, A549, and HCC1588) and normal lung cells (MRC5) assessed by Immunoblotting. (C) The levels of SIRT3 and FOXO3 and ace‐FOXO3 level assessed by Immunoblotting after A549 cells were infected with lentiviral vectors containing oe‐SIRT3 or oe‐NC, sh‐FOXO3 + DMSO or sh‐FOXO3 + MG132 *p < 0.05 vs. adjacent normal tissues, MRC5 cells or oe‐NC; #*p* < 0.05 vs. sh‐FOXO3 + DMSO. The unpaired *t*‐test was applied for comparisons between two groups

As shown in Figure 4A, RT‐qPCR and Immunoblotting results displayed that mRNA and protein expression of SIRT3 was strikingly inhibited in lung cancer tissues compared with adjacent normal tissue.

To further confirm whether FOXO3 is regulated by SIRT3 at acetylated level, we adopted Immunoblotting displayed that compared with normal lung cells, reduced level of SIRT3 and increased ace‐FOXO3 level were observed in lung cancer cells (Figure 4B). Moreover, A549 cells were infected with lentiviral vectors containing oe‐SIRT3 or oe‐NC. Immunoblotting results demonstrated that SIRT3 overexpression elevated level of FOXO3, but reduced ace‐FOXO3 level compared with oe‐NC treatment. MG132 treatment rescued reduction in FOXO3 level caused by sh‐FOXO3. Coherently, SIRT3 elevation inhibited FOXO3 at acetylated level, thus, elevating FOXO3 expression (Figure 4C).

### 3.5 SIRT3 modulates FOXO3/CDT1 axis to enhance cisplatin resistance of lung cancer cells

**FIGURE 5:**
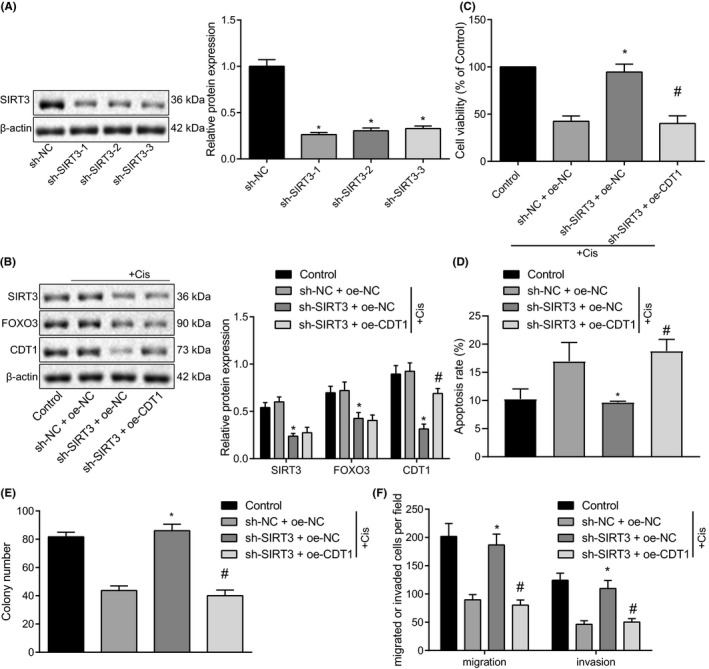
SIRT3 overexpression elevates FOXO3/CDT1 axis to enhance sensitivity of lung cancer cells to cisplatin. (A) The silencing efficiency of SIRT3 validated using Immunoblotting. (B) The protein expression of SIRT3, FOXO3, and CDT1 determined using Immunoblotting. (C) The viability of A549 cells determined using CCK‐8. (D) The apoptosis of A549 cells determined using flow cytometry. (E) The proliferation of A549 cells assessed using colony formation assay. (F) The invasion of A549 cells following CDT1 overexpression determined using Transwell. *p < 0.05 vs. sh‐NC; #*p* < 0.05 vs. sh‐SIRT3 + oe‐NC. The unpaired *t*‐test was applied for comparisons between two groups. One‐way ANOVA was used for comparisons among more than two groups, followed by Tukey's post hoc test

We downregulated SIRT3 in A549 cells and silencing efficiency was validated using Immunoblotting. The sh‐SIRT3‐1 with most significantly suppressed SIRT3 expression was selected for subsequent analysis (Figure 5A). Meanwhile, lentiviral vectors containing sh‐SIRT3 + oe‐CDT1 was transduced into A549 cells (Figure 5B). A series of procedures including CCK‐8, flow cytometry, colony formation assay, and Transwell results revealed that sh‐SIRT3 + oe‐NC treatment effectively promoted viability, proliferation, and invasion, but inhibited apoptosis of A549 cells compared with sh‐NC + oe‐NC treatment, but this trend was reversed by CDT1 overexpression (Figure 5C–F), suggesting enhanced sensitivity of lung cancer cells to cisplatin. Taken together, SIRT3 regulates FOXO3/CDT1 axis, thus enhancing cisplatin resistance of lung cancer cells.

### 3.6 SIRT3 elevation inhibits cisplatin resistance of lung cancer cells through FOXO3/CDT1 axis *in vivo*

**FIGURE 6:**
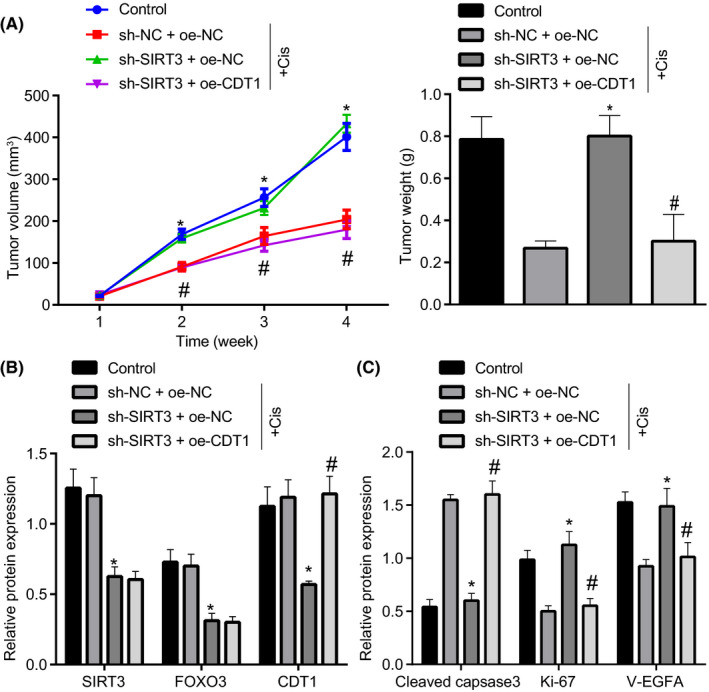
SIRT3 elevation suppresses cisplatin resistance of lung cancer cells through FOXO3/CDT1 axis *in vivo*. (A549 cells transduced with lentiviral vectors containing sh‐NC + oe‐NC, SIRT3 + oe‐NC, or sh‐SIRT3 + oe‐CDT1 were injected in cisplatin‐treated nude mice). (A) The tumor volume and weight extracted from nude mice xenografted with tumor. (B) The SIRT3, FOXO3, and CDT1 protein expression determined using Immunoblotting. (C) The protein expression of cleaved caspase3, Ki‐67, and VEGFA determined using Immunoblotting. *p < 0.05 vs. sh‐NC + oe‐NC; #*p* < 0.05 vs. sh‐SIRT3 + oe‐NC. The unpaired *t*‐test was applied for comparisons between two groups. One‐way ANOVA was used for comparisons among more than two groups, followed by Tukey's post hoc test

A549 cells transduced with lentiviral vectors containing sh‐NC + oe‐NC, SIRT3 + oe‐NC, or sh‐SIRT3 + oe‐CDT1 were injected in cisplatin‐treated nude mice, and a group of blank controls mice without cisplatin treatment were established. The tumor volume and weight were recorded. As expected, sh‐SIRT3 + oe‐NC treatment strikingly promoted tumor volume and weight relative to sh‐NC + oe‐NC, which was reversed by CDT1 overexpression (Figure 6A). Moreover, Immunoblotting results of SIRT3, FOXO3, and CDT1 expression were consistent with that in A549 cells (Figure 6B). Subsequently, Immunoblotting results demonstrated sh‐SIRT3 + oe‐NC treatment declined levels of cleaved caspase3, but elevated Ki‐67 and VEGFA levels in cisplatin‐treated mice, whereas trend was reversed by CDT1 overexpression (Figure 6C). Taken together, SIRT3 elevation inhibits cisplatin resistance of lung cancer cells through FOXO3/CDT1 axis *in vivo*.

## 4 DISCUSSION

**FIGURE 7:**
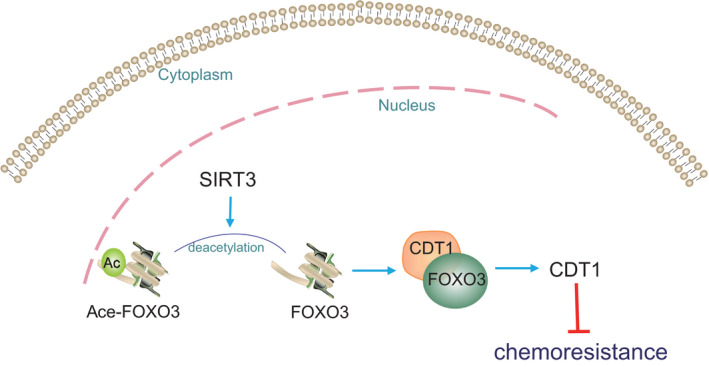
The role of SIRT3/FOXO3a/CDT1 axis in cisplatin resistance of lung cancer

The long‐term use of cisplatin is associated with enhanced enhance drug resistance, which consequently debilitates therapeutic efficacy and clinical outcome.^14^ Recently, a prior study noted that SIRT3 increases SCLC chemosensitivity by promoting cell apoptosis by reducing p53 expression.^15^ In the current study, we investigated underlying mechanism of SIRT3 in cisplatin resistance in lung cancer. Our findings support the hypothesis that overexpression of SIRT3 elevates FOXO3a/CDT1 axis, thus contributing to inhibited lung cancer cell viability, proliferation, and invasion as well as enhanced cell apoptosis and sensitivity of lung cancer cells to cisplatin (Figure 7).

An existing study documented rapid proteolysis of CDT1 was induced by cisplatin treatment in human cervical carcinoma HeLa and hepatocarcinoma HepG2 cells.^16^ The expression of CDT1 was deregulated by administration of hexavalent chromium (a well‐known carcinogen) treatment in lung cancer.^17^ Poorly expressed CDT1 has been reported to be evident following Cul4A overexpression‐induced transgenic mouse model of lung tumorigenesis, and Cul4A depletion contributed to increased sensitivity to chemotherapy drug cisplatin by elevating CDT1 expression in lung cancer cells.^18^ Existing studies were consistent with our findings supporting that CDT1 was repressed in lung cancer tissues and cells, and its elevation fundamentally contributed to inhibited cisplatin resistance of lung cancer cells. Moreover, the increased proportions of Ki‐67^+^ proliferating cells and upregulated expression of VEGFA in lung cancer were associated with stimulated cell proliferation and angiogenesis, respectively.^19^ In current study, we identified that upregulated CDT1 contributed to reduced Ki‐67 and VEGFA levels, indicative of inhibited cell proliferation and angiogenesis *in vivo*.

In subsequent experiments, we found that FOXO3 positively regulated CDT1 expression, and its elevation inhibited cell cisplatin resistance of lung cancer cells as well as inhibited viability, proliferation. Consistently, FOXO3 expression was depleted in human lung adenocarcinoma, and its activation upregulated level of caspase‐dependent apoptosis in lung adenocarcinoma cells exposed to this DNA‐damaging carcinogen.^20^ The depletion of FOXO3 was consequent for increased promoted susceptibility to bleomycin challenge, accompanied by augmented fibrosis and loss of lung function in idiopathic pulmonary fibrosis. Another study highlighted that suppression of FOXO3 targeted by miR‐551b boosted cell proliferation, invasion, and cisplatin resistance of ovarian cancer. The FOXO3 elevation could potentially invert the cisplatin resistance in ovarian cancer.^21^

Another important finding of the current study established that is that SIRT3 elevation inhibits FOXO3 at the acetylation level, thus, elevating and concentrating FOXO3 expression. In consistency with our study, geminin‐associated HDAC3 has been proposed to deacetylate FOXO3 to inhibit its transcriptional activity, thus inhibiting downstream FOXO3 target Dicer, an essential RNase to inhibit metastasis of breast cancer cell.^22^ In context of hypoxia, SIRT3 elicited functionality as a stabilizer of FOXO3 *via* deacetylation, thus, increasing adaptive capacity of endothelial cells.^23^ Furthermore, our findings revealed that SIRT3 elevation inhibited cell cisplatin resistance of lung cancer cells. Consistent with our study, activation of SIRT3 promoted sensitivity of ovarian cancer cells to cisplatin regulated by ABT737.^24^ Moreover, enforced level of apoptosis‐related protein cleaved caspase 3 was correlated with promoted apoptosis in NSCLC.^25^ Concordantly, in current study, elevated Ki‐67 and VEGFA levels, but downregulated cleaved caspase 3 level was induced by treatment of SIRT3 depletion, which was reversed by OCD1 elevation *in vivo*.

To sum up, current investigation supported hypothesis demonstrating role of SIRT3 in inhibiting resistance of lung cancer cells to cisplatin. Mechanically, overexpressed SIRT3 elevates expression of FOXO3a/CDT1 axis, thus, contributing to enhanced cell apoptosis and sensitivity of lung cancer cells to cisplatin. Importantly, reported observation suggested a novel mechanism of cisplatin resistance involving transcription factors and mRNAs in lung cancer. However, we cannot exclude involvement of other signaling pathways in cisplatin resistance of lung cancer cells regarding reported axis due to complex microenvironments, which warrants further exploration in future.

## CONFLICT OF INTEREST

The authors declare no conflict of interest.

## Data Availability

The data sets generated and/or analyzed during the current study are available from the corresponding author on reasonable request.
